# Physiologically Realistic and Validated Mathematical Liver Model Revels Hepatobiliary Transfer Rates for Gd-EOB-DTPA Using Human DCE-MRI Data

**DOI:** 10.1371/journal.pone.0095700

**Published:** 2014-04-18

**Authors:** Mikael Fredrik Forsgren, Olof Dahlqvist Leinhard, Nils Dahlström, Gunnar Cedersund, Peter Lundberg

**Affiliations:** 1 Wolfram MathCore AB, Linköping, Sweden, and Center for Medical Image Science and Visualization (CMIV), Linköping University, Linköping, Sweden; 2 Department of Radiation Physics and Division of Radiological Sciences, Department of Medical and Health Sciences, Linköping University, Linköping, Sweden; 3 Center for Medical Image Science and Visualization (CMIV), Linköping University, Linköping, Sweden; 4 Department of Radiology and Division of Radiological Sciences, Department of Medical and Health Sciences, Linköping University, Linköping, Sweden; 5 Department of Experimental Medicine, Linköping University, Linköping, Sweden; 6 Systems Biology, Department of Biomedical Engineering, Linköping University, Linköping, Sweden; 7 Department of Radiation Physics, Department of Radiology and Division of Radiological Sciences, Department of Medical and Health Sciences, Linköping University, Linköping, Sweden; Heidelberg University, Germany

## Abstract

**Objectives:**

Diffuse liver disease (DLD), such as non-alcoholic fatty liver disease (NASH) and cirrhosis, is a rapidly growing problem throughout the Westernized world. Magnetic resonance imaging (MRI), based on uptake of the hepatocyte-specific contrast agent (CA) Gd-EOB-DTPA, is a promising non-invasive approach for diagnosing DLD. However, to fully utilize the potential of such dynamic measurements for clinical or research purposes, more advanced methods for data analysis are required.

**Methods:**

A mathematical model that can be used for such data-analysis was developed. Data was obtained from healthy human subjects using a clinical protocol with high spatial resolution. The model is based on ordinary differential equations and goes beyond local diffusion modeling, taking into account the complete system accessible to the CA.

**Results:**

The presented model can describe the data accurately, which was confirmed using chi-square statistics. Furthermore, the model is minimal and identifiable, meaning that all parameters were determined with small degree of uncertainty. The model was also validated using independent data.

**Conclusions:**

We have developed a novel approach for determining previously undescribed physiological hepatic parameters in humans, associated with CA transport across the liver. The method has a potential for assessing regional liver function in clinical examinations of patients that are suffering of DLD and compromised hepatic function.

## Introduction

Diffuse liver disease is a rapidly growing problem throughout the Western world. The pathology include conditions such as viral hepatitis C (prevalence of about 1.8–3.2% [1], [2]), and non-alcoholic fatty liver disease (prevalence of about 16–20% [2], [3]), which all can provoke the formation of fibrosis, inflammation and ultimately, cirrhosis. In the final stages of these diseases there is a loss of liver function, and a reliable evaluation of liver function is crucial, for example, for the management and planning of liver resection or transplantation, which may be the only available treatment in severe liver disease. Liver function is often assessed using methods such as Indocyanin-Green 15 retention rate (ICGR15) or Tc-99m galactosyl human serum albumin (GSA) measurements [4]–[6]. ICGR15 and GSA are both exclusively global indicators, *i.e.* they do not provide any information about regional liver function. In addition, GSA involves the injection of a radioactive isotope, and is therefore associated with certain risks as well as costs. These issues can all be overcome by use of magnetic resonance imaging (MRI) and MRI contrast agents (CA), providing a local completely non-invasive assessment of liver function, without the use of ionizing radiation.

MRI is already an established diagnostic modality, but the techniques and CA are under constant development. One recently approved novel CA is Gd-EOB-DTPA (abbreviated as ‘EOB’ in the following text; Primovist/Eovist, Bayer Schering Pharma, Berlin, Germany). EOB is particularly advantageous for the study of liver function for several reasons. One is that EOB is specifically taken up from plasma by hepatocytes, followed by a subsequent active excretion into the bile by the hepatocytes. Typically, the livers of healthy subjects excretes about 50% of the injected EOB dose into the bile, an absorption/excretion rate which is much higher than an alternative CA, Gd-BOPTA [7]–[9]. In addition, EOB appears to use the same mechanisms for cellular uptake and release as the commonly used ICG [6]. Consequently, EOB-enhanced hepatobiliary MRI has a clear potential to become a valuable standard diagnostic technique in the clinical workflow [4], [10]–[12], although the methods for the data analysis needs to be considerably improved before that can materialize.

In previous human studies with dynamic contrast-enhanced MRI (DCE-MRI) using EOB the methods for estimating liver function has been based on analyzing deconvoluted liver response, based on input functions estimated in the portal vein. This liver response was then analyzed using methodology developed for scintigraphy in order to estimate the hepatic extraction fraction [13]. A more sophisticated method was recently published, combining hepatic perfusion with functional modeling, where the dual blood supply to the liver was taken into account, as well as a more refined measure of the hepatocyte uptake kinetics [14]. This later method unfortunately requires a high temporal resolution, which in turn implies a lower spatial resolution, which will affect the signal-to-noise ratio, and therefore will render the image matrix and field of view incompatible with diagnostic imaging of *e.g.* focal lesions. Both of the above mentioned methods demand an input function estimated from a single vessel in order to calculate the kinetic parameters. Moreover, the non-linear relationship between EOB concentration and microenvironment (*e.g.* blood plasma, hepatocyte) as well as the experimental parameters are not taken into account. A novel, recently published method for quantification of signal enhancement in the images allows for rescaling of the signal intensities according to the specific experimental setup and microenvironment [10]. This method allows for our here proposed approach, based on novel principles derived from systems biology, and was recently successfully used to estimate EOB concentrations from DCE-MRI data for modeling the CA-uptake in rats [15].

In systems biology, data analysis is entirely focused on biologically relevant mechanistic mathematical models [16], [17]. These mathematical models are formal representations of mechanistic hypotheses regarding how the data have been generated. Models that are unable to describe the data sufficiently well lead to rejected hypotheses, whereas non-rejected models can be used for instance for the identification of mechanistic parameters, and importantly other not directly measurable properties in the system. Such a systems biology approach has been used successfully on a wide variety of different systems [18], [19], but no mechanistic mathematical model for human liver function assessment based on high spatially resolved EOB DCE-MRI data has yet been developed.

The main aim of this work was to develop a physiologically based whole body mathematical mechanistic minimal model with a potential for assessing liver function based on DCE-MRI of a liver-specific CA (EOB), in humans. Furthermore, we intended to combine the modeling with a previously developed approach for MRI-signal based CA concentration quantification [10] in order to allow for physiologically relevant and quantitative measures. In addition, we wished to develop an approach that is compatible with routine clinical protocols, allowing for morphological and regional evaluation of liver status.

## Materials and Methods

### Experimental Data

The experimental data was obtained from two previously published reports, no experimental data was produced. The retrospective study included healthy volunteers from Dahlqvist Leinhard *et al* [10] (*n* = 10; referred to as ‘estimation data’), and a preclinical study by Schuhmann-Giampieri *et al* [8] (*n* = 18; referred to as ‘validation data’). These two studies differ in the measurement technique as well as injection procedures which make this a particularly useful validation set in terms of validating that this is a physiologically sound model applicable on a wide range of conditions. The most relevant experimental parameters of the two studies are cited and listed in Table 1. All images and other relevant data in the estimation data set were available to us, whereas the validation data were extracted from the printed article.

**Table 1:** Experimental data description.

	Estimation data – Dahlqvist Leinhard *et al* [10]	Validation data – Schuhmann-Giampieri *et al* [8]
Injection procedure	Bolus	Infusion
Injection rate	1 mL·s^−1^	5–20 mL·s^−1^
Injection time	<15 s	10 min
EOB dose	0.025 mmol·kg^−1^·BW^−1^	0.2, 0.35, 0.5 mmol·kg^−1^·BW^−1^
# subjects	10	6 in each dose-group (18 in total)
Mean age	25 (22–27) years	30 (20–40) years
Mean weight	73 (62–84) kg	83 (69–97) kg
Gender	5 males, 5 females	18 males
Measurement technique	DCE-MRI^a^	ICP-AES^b^
Measurement time	Pre-contrast, arterial and portal venous phase, 10, 20, 30 and 40 min	0–120 h, 6 days^c^
Measurement	ROIs in the liver, spleen, veins	Sampling of blood, feces, and urine

a Dynamic Contrast Enhanced Magnetic Resonance Imaging.

b Inductively Coupled Plasma Atomic Emission Spectrometry.

c Blood sampling up to 120 h, only data points up to about 40 min post-contrast was used in the validation thus matching the time-span of the DCE-MRI data. Feces and urine sampled up to 6 days.

The study from which the estimation data was obtained was approved by the regional ethical review board in Stockholm, Sweden, (‘Regionala etikprövningsnämnden i Stockholm’; Reference No. EPN 2005/305–31/1) and the participants gave informed written consent.

### DCE-MRI Signal Intensity Conversion of the Estimation Data

**Figure 1:**
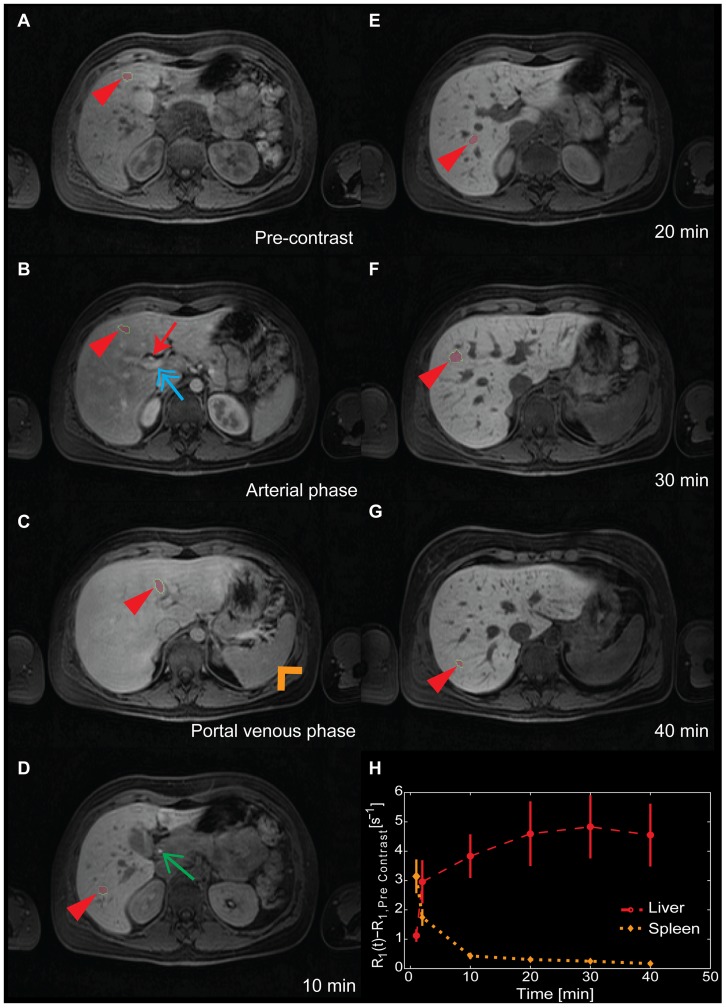
Estimation data. Example of the spatially highly resolved DCE-MRI that was used as estimation data, showing the pre-contrast acquisition (panel A), followed by the post-contrast acquisitions (panels B–G) with a distinct accumulation of the contrast agent in the liver. The dark areas within the liver in the late phases (panels D–G) are mainly blood vessels with drastically lower concentration of contrast agent compared to the accumulated contrast agent in the hepatocytes. This difference can be appreciated quantitatively in panel H, since the increase in signal intensity in the spleen is only due to the CA residing within the blood plasma. The location of the seven different regions of interest placed in the liver are shown throughout the time series (panels A–G, one in each panel), indicated by the red arrow heads. The hepatic artery, with a high initial concentration of contrast, can be seen in panel B (red arrow) as well as the portal vein (blue double arrow). In panel C the spleen (orange open arrow head) is almost isointense with the liver. The efflux of CA in the common bile duct can be seen in panel D (green arrow). Finally, the quantified mean relative change in relaxivity in the ROIs placed in the liver and spleen throughout the examination is shown in panel H, with the vertical bars corresponding to the standard error of the mean.

Regions of interest (ROI) where placed in the images by an experienced radiologist. Seven ROIs were placed within the liver, three within the spleen and one each in the portal and splenic vein. The seven liver ROIs were placed in both the left and right liver lobes avoiding any large vessels or focal lesions, but without the intention of strictly following the segmental division as introduced by Couinaud [20]. The sizes of the ROIs where arbitrarily chosen by the radiologist, but adjusted to be equal in size and approximate position throughout the time series. Landmarks in the images where used for correcting movement of the liver between the acquisitions. The signal intensity in the ROIs were subsequently converted into quantitative CA concentrations as previously described by Dahlqvist Leinhard *et al* [10]. Fig. 1A–D shows an example of how seven different liver ROIs were placed in one subject throughout the volume in a spatially highly resolved DCE-MRI time series. In Fig. 1E the quantified mean of all ROIs placed in the liver as well as in the spleen are shown.

### Mathematical Models

The model was formulated using a system of ordinary differential equations (ODEs) using the following notation,
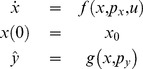
(1)where 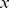 denotes the states (here corresponding to CA concentration in different compartments) and the dot 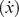 denotes time-derivative; 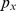 denotes kinetic transport parameters and volumes; 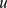 denotes the injection of CA; 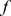 and 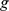 are smooth nonlinear and linear continuous functions; 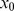 denotes the initial state values; 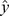 denotes the model output corresponding to measurements; and 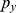 denotes the parameters used to calculate the simulated output from the states (compartmental fractions and scaling parameters). Note that all symbols are vectors and that the time-dependence of 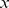, 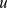 and 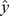 usually are dropped to simplify the notations. The function 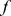 was constructed after summation of in- and out-going flows for each compartment, according to normal conventions [16], [21]. Finally, specific choices for the equations in Eq. 1 corresponds to a specific model structure *i* (denoted M*i, e.g.* M0), and a model structure with specific values for the parameters corresponds to a specific model (denoted M*i*(*p*)).

### Modeling Software

The models were implemented and analyzed in the ‘Systems Biology ToolBox2’ (SBTB2) v2.1 for MATLAB [22] (obtained from www.sbtoolbox2.org); SBTB2 is a free open-source add-on package to MATLAB (R2009b, The MathWorks inc., MA, U.S.). All relevant scripts and model files used for the analysis are available at the journal home page (File S1).

### Identification of Model Parameters

The model simulations were compared to the experimental data by evaluation of the size of the residuals, which were defined as the difference between the measured 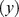 and simulated 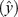 outputs. If these residuals are normalized by the standard deviation 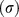 of the measurement uncertainty, the following cost function measures the overall agreement between model and data.
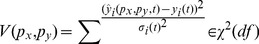
(2)where the summation is over *t* and *i*, and where the final 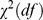 symbol is described in relation to the statistical tests below, and the cost 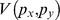 depends on the parameter values [16]. Since the specific parameter values are not known, an optimization procedure was used to identify those parameters that gave the lowest cost, *i.e.*, the best agreement with the data. A simulated annealing [23] approach, modified to return not only the optimal parameters but all parameters that passes the chi-square test (Eq. 2, see description of the test below), was used for the optimization.

### Model Analysis

The estimated parameters were then analyzed in three ways. First, one probes whether the agreement to the data is sufficient. This was formally evaluated using a chi-square test, which in practice tested whether the cost function (Eq. 2) was lower than a certain threshold, 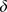. This threshold was calculated as the inverse of the cumulative chi-square distribution, where the significance level was chosen as 0.05, and where the degrees of freedom *df* were chosen as the number of data-points (18 for estimation data used in the optimization, and 9 for each set of validation data) minus the number of identifiable parameters (4 for estimation data, and 0 for validation data) [16]. Non-rejected models were passed on to the remaining two steps in the model analysis.

The second test concerns parameter identifiability. A parameter is (practically) identifiable if its value can be determined from data with a reasonably small uncertainty. This identifiability analysis was carried out using the Profile Likelihood (PL) approach [24]. PL analyses is done one parameter at a time, by increasing (or decreasing) that parameter while optimizing all other parameters; until the parameter has increased (or decreased) so much that the optimization of the other parameters no longer can produce an acceptable agreement with the data, the upper (lower) boundary for that parameter has then been found.

The third test concerns predictions. In this step, we translated the uncertainty in the parameters to an uncertainty in the predictions. This was implemented by using the core prediction methodology introduced in [25]. The basic idea is to determine a representative subset of all acceptable parameters (here obtained in the PL analysis), and then observing the combined simulations of the entire parameter set. Herein two different aspects of the model were evaluated using the core prediction methodology: 1) the amount of EOB eliminated via the bile and urine and 2) the amount of EOB residing within the blood plasma. A benefit of this approach is that it, unlike most other approaches, it also works in the case of unidentifiable parameters.

Furthermore, the sensitivity of the predictions was evaluated by simulating the final model using a ±20% variation, in each individual volume parameter at a time. Literature derived values for each volume parameter were thus varied in the final model to determine the sensitivity of the results associated witch such variation.

### Model Description

**Figure 2:**
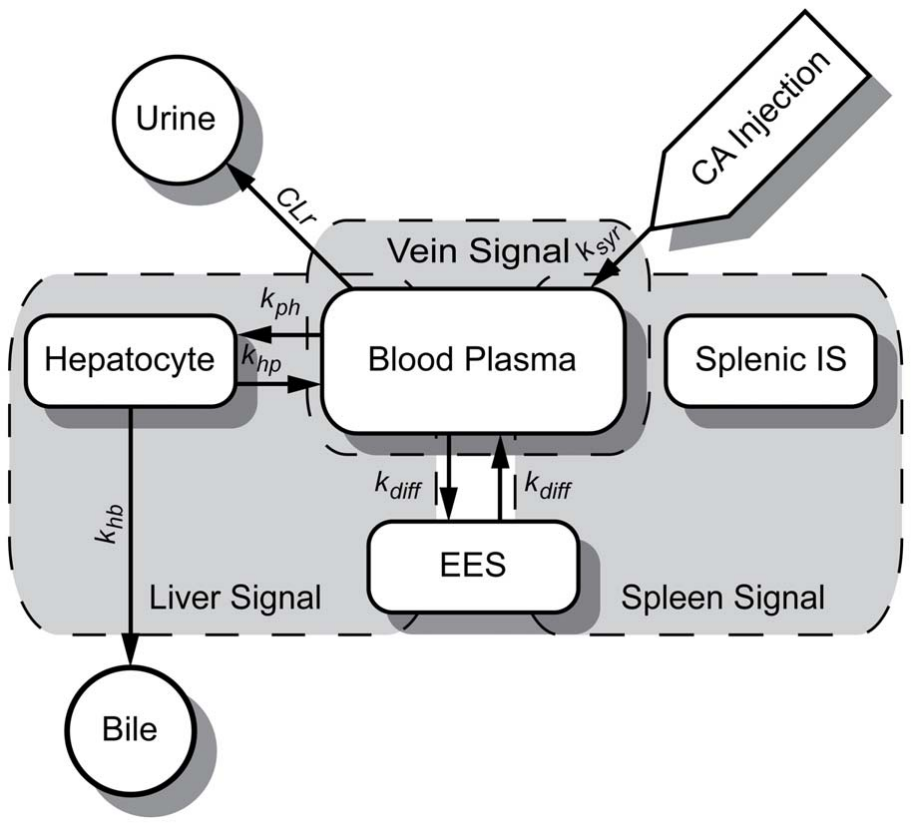
Diagram of the proposed model. Schematic diagram of the proposed model (M0). The *rounded edge rectangles* represent the different compartments in the model connected with *arrows* showing the direction of the CA fluxes, with the associated rate parameters. The Hepatocyte compartment represents all intracellular water within the liver, and the Splenic IS represents the structures within the spleen that are inaccessible to EOB. The *white pointed rectangle* (CA Injection) represents the administration of EOB to the system. The *white circle*s are the sinks of the system, meaning that once the CA has reached the ‘Urine’ and ‘Bile’ compartments it cannot be transferred back into the system. The signal model is represented by the *dashed grey boxes* (liver, vein and spleen respectively), showing the congregation of compartments for the conversion from CA concentration to relative change in relaxivity that is comparable to the data from the ROIs. The fractions of the compartments used in each MRI signal simulation can be found in Table 2. Both the plasma and EES compartments are shared between the liver and spleen MRI signals.

**Figure 3:**
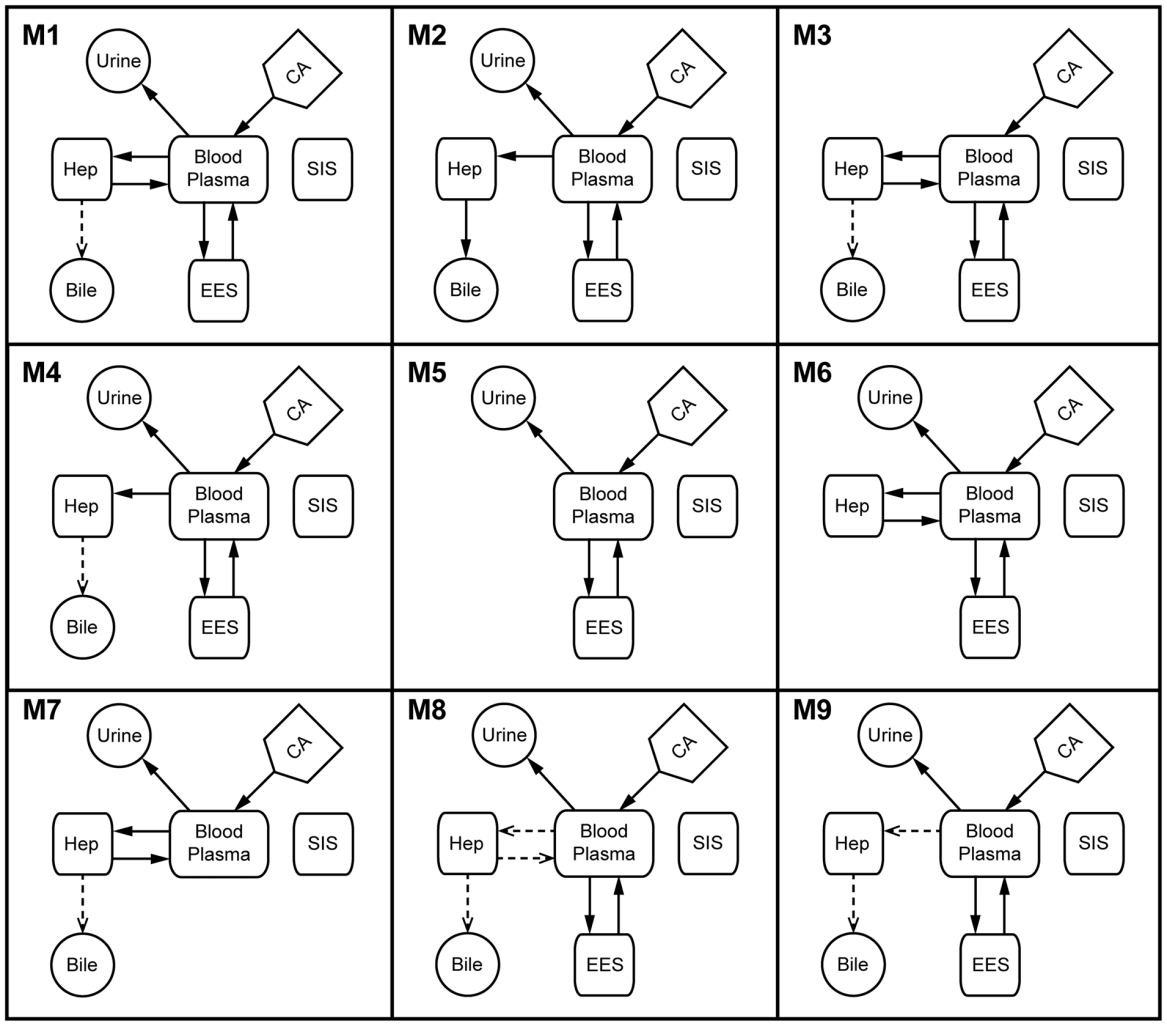
Overview of all tested model configurations. Schematic diagram of the rejected model variants (M1–M9), where the *squares* represents compartments, *circles* are the sinks in the model, and the *arrows* shows the fluxes in the model (see Fig. 2). The signal model (see Fig. 2) was omitted for simplicity and it is identical in all models (M0–M9). The *dashed arrows* represent the use of Michaelis-Menten kinetics instead of linear mass-action or diffusion like kinetics (as shown by *solid arrows*).

Several different model structures were analyzed in detail, even though only a single model is ultimately suggested. The suggested model structure is denoted M0, and additional proposed model structures are denoted M1–M9. M0 is depicted in Fig. 2 and corresponding figures for the other model structures are found in Fig. 3. Fig. 2 contains four rounded-edge rectangles, and these correspond to the four major compartments in the model: ‘Blood Plasma’, ‘Extracellular Extravascular Space’ (EES), ‘Splenic Intracellular Space’ (Splenic IS), and ‘Hepatocytes’ (corresponding to concentrations 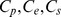 and 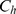 respectively). The two circles, ‘Bile’ and ‘Urine’, correspond to sinks in the model, *i.e.* there is no flow going from these compartments to other parts of the model. The shaded areas correspond to the three measurement signals. The leftmost signal in Fig. 2 is the MRI ‘Liver Signal’, which is composed of contributions from the Hepatocytes, Blood Plasma and EES. Similarly, the MRI ‘Spleen Signal’ has contributions from Splenic IS, Blood Plasma, and EES, and the MRI ‘Plasma Signal’ emerges from the Blood Plasma compartment only. The arrows correspond to fluxes, *i.e.*, transport between the compartments, and the different model versions differ from each other in the characterization of these fluxes.

### Transports

The first transport step is the injection of CA. This flux is modeled as a step function
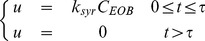
(3)where 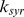 corresponds to the CA injection rate, 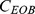 corresponds to EOB concentration in the syringe, and 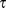 corresponds to the time point where the injection is terminated. This transport equation is the same for all different models, and in the case of using the model with the validation data, these parameters were updated in order to match the experimental setup.

There is no transport of EOB between Splenic IS and the other compartments; similarly this is the same for all different models.

There is a bi-directional flow between blood plasma and EES. This flow is caused by passive diffusion, where the diffusion constant is the same in both directions
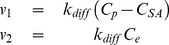
(4)where 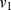 and 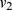 corresponds to the flow to and from EES, respectively; where 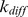 is the diffusisssson rate constant; and where 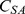 is the concentration of EOB bound to serum albumin [26]. 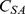 is given by 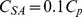. Note that 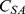 is located in the plasma compartment, therefore it is a part of the plasma EOB (*i.e.* contributes to 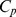); in other words, 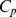 consists of both free and bound EOB. Eq. 4 describes the flow between Plasma and EES for all model structures, with the exception of M7.

The flow between the blood plasma and hepatocytes is governed by different membrane associated macromolecular transporters, mainly, OATP1B3, OATP1B1, and also by NTCP [27], [28]. Back-flow into the blood plasma from the hepatocytes can be facilitated by a number of different routes; 1) primarily the OATP's due to their function as bidirectional transporters [29], [30], 2) up-regulation of MRP3 on the sinusoidal membrane [31], and in some diseased states 3) tight-junction leakage [32]. The flows between Blood Plasma and Hepatocyte compartments were, due to the multiple transporters, modeled in a phenomenological manner: as linear mass-action transports in M0–M4, M6, and M7, alternatively as Michaelis-Menten expressions in M8 and M9 (Fig. 3). Furthermore, omitted in M5 and in some model structures (M2, M4, and M9) the flow was modeled as irreversible, *i.e.* where there is no flow from the hepatocytes to the blood. In other words, for M0, the flows are given by:
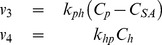
(5)where 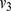 and 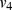 corresponds to the flow to and from the hepatocyte, respectively, 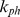 and 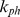 are the kinetic parameters for the two rate equations.

The blood plasma is also cleared of CA by the kidneys. This clearance primarily consists of glomerular filtration, which results in an apparent clearance of approximately 118 mL of blood per minute [7], [8], [33], which means that the renal clearance can be modeled as
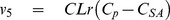
(6)where 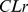 is the kinetic parameter.

The final transport in the model is from the hepatocytes to the bile, which is mediated via MRP2. This transport is generally believed to be rate limiting, displays Michaelis-Menten kinetics, and it is an ATP-driven strictly unidirectional process [34]–[36]. For these reasons, the flow is in most models described by Michaelis-Menten kinetics (Eq. 7b M1, M3–M4, M7–M9), but in model M0 it was shown possible to use a linear expression instead (Eq. 7a). Note that we in this work are seeking a minimal description of the system with identifiable parameters given the data available, therefore even though there is evidence for saturation in this particular transporter arguing for the use of a Michaelis-Menten rate expression, a linear approximation might nevertheless be sufficient when the transporter is included in the complete system, *e.g.* by using mass action-like behavior instead [37]. See the discussion for more detailed motivation for such replacement of Michaelis-Menten with mass action.
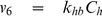
(7a)

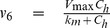
(7b)where 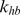 is the kinetic parameter for EOB transfer from the hepatocytes to the bile canaliculi. Also note that in M6, this flux (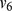) is omitted (Fig. 3).

By including these flows, the differential equations become:
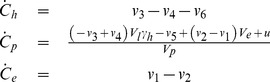
(8)


These ODEs are the same for all model structures, even though some flows, 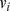, are equal to zero in some of the models.

### Signal Model

The signal model depended on whether we compared with estimation data (derived from DCE-MRI) or validation data (derived from blood sampling). For the validation data, the measurement equation was simply 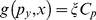, where 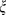 is an arbitrary scaling constant. For the estimation data, the conversion of contrast agent concentrations in the model to relative change in relaxivity rested on the following relationship [10]:
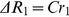
(9)


As described above, each ROI is an observation of the combined signal from multiple compartments, *e.g.* the MRI Liver Signal contains contributions from EES, Blood Plasma and the Hepatocyte compartments. In order to derive a value comparable to the ROIs placed in the tissue, the following equation was used, based on Eq. 9:
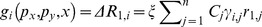
(10)where 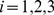 for the three measurements in the liver, spleen and veins, respectively; where 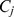 is the CA concentration in compartment *j*, for the four compartments (Hepatocyte, EES, Splenic IS, or Blood Plasma); where γ*_i,j_* is the fraction of the volume in ROI *i* consisting of compartment *j* and r_1,*j*_ is the *in situ* relaxivity in compartment *j*. The *in situ* compartment specific relaxivity values at 1.5 T and 310 K were assumed to be: EES 6.9 mmol^−1^·s^−1^, Blood Plasma 7.3 mmol^−1^·s^−1^, and Hepatocytes 10.7 mmol^−1^·s^−1^ [38], [39].

### Published Parameter Values

Some of the parameters were estimated using optimization, as described above, whereas other parameters were obtained from literature. In Table 2, the individual fractions, γ*_i,j_* (used in Eq.10) are given. These fractions are based on a 70 kg ‘standard human’, characterized by 20% ‘fat’ (v/v) [40]. Note that the fractions γ*_i,j_* for the splenic ROIs do not add up to zero. The reason is that the cells of the spleen were not modeled because they do not take up any observable amounts of CA.

**Table 2:** Fraction parameters defining the content in each ROI.

Compartment	Fraction of Liver ROI	Fraction of Spleen ROI	Fraction of Vessel ROI
Blood Plasma	0.12	0.35	1.0
EES	0.20	0.20	
Hepatocyte	0.68		

The fractions (γ in Eq. 8 and Eq. 10), defining which compartment, and how much of each compartment is included in measured regions of interests. This is shown graphically in Fig. 2, the signal model. The fractions were obtained from [40].

The volumes were also assumed to be known, and they were also obtained from the ‘standard human’ [40]: 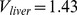, 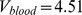 and 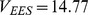 [40]. Since the CA does not enter the erythrocytes the blood plasma volume is also needed. Assuming an average hematocrit of 0.43, the blood plasma volume in a typical human subject is 1.94 L [40].

## Results

### Model Fit of DCE-MRI

**Figure 4:**
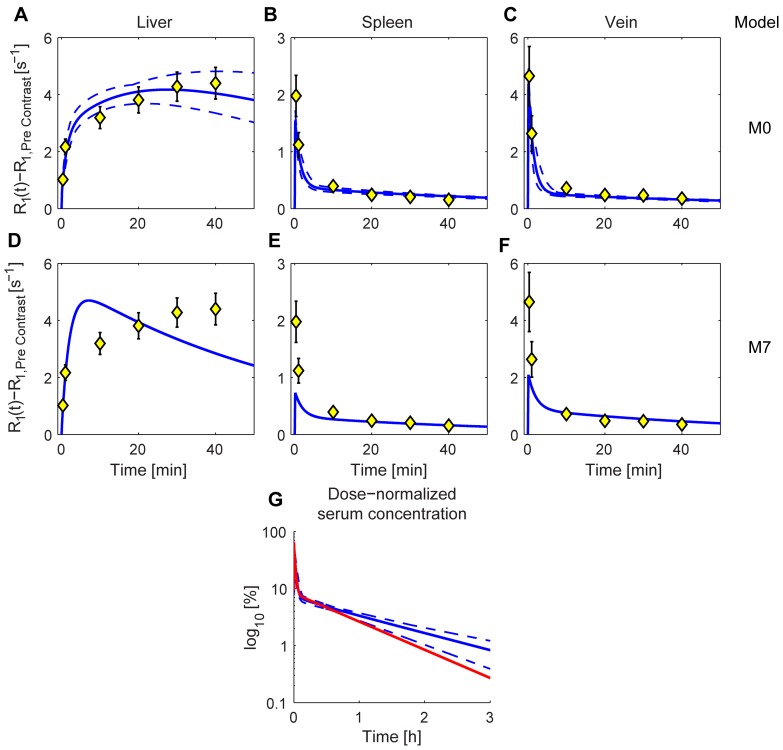
Predictions after model fitting for the proposed and one rejected model. Panels A–F shows model prediction versus estimation data (black error bars, presented as mean ± SE). Panels A–C shows the predicted MRI signals (solid blue lines) for the liver, spleen, and vein respectively using model MO. The dashed blue lines indicate the range of the predictions defined by the profile likelihood–based confidence intervals of the parameters (Table 3, Fig. 5). Panels D–F shows the corresponding simulations for a rejected model, in which diffusion between the blood plasma and the EES was assumed to be zero (model M7). In panel G, the dose-normalized blood plasma CA concentration is shown for model M0 (blue lines), and also the prediction with the lowest cost that failed to pass the *ad hoc* constraint (red line).

**Figure 5:**
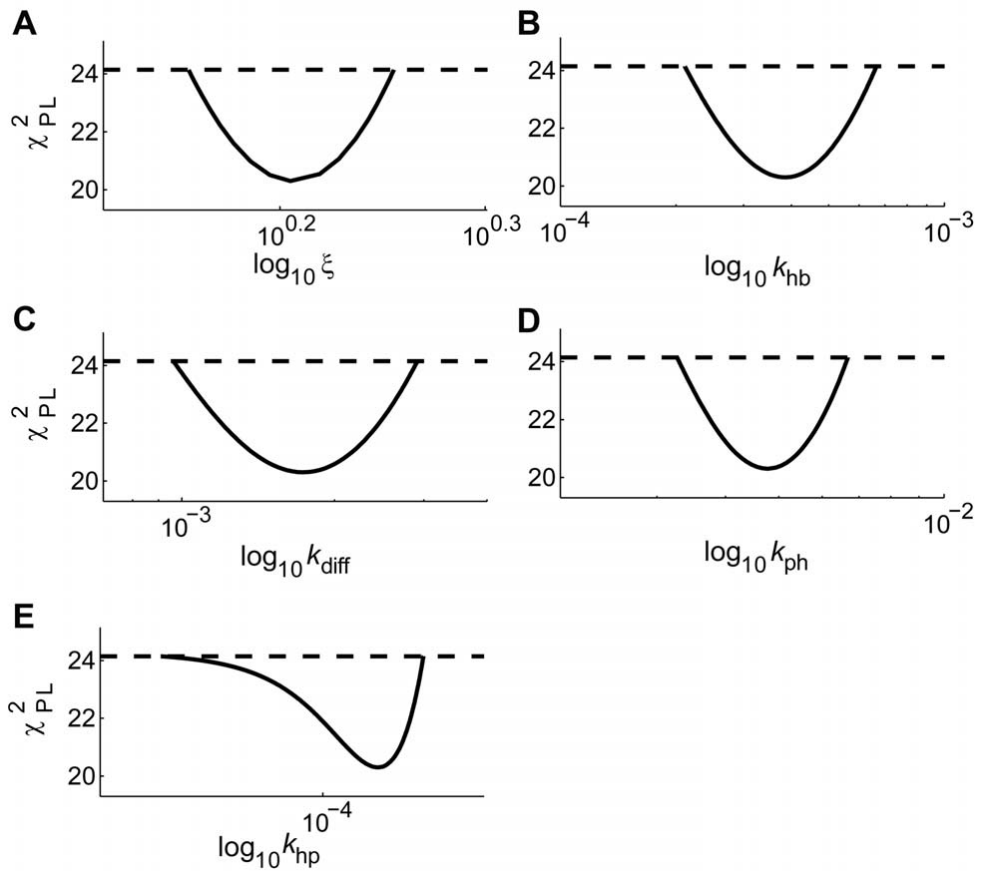
Profile likelihood parameter uncertainties for the proposed model. The solid lines show the profile likelihood versus parameter value for model M0, given the estimation data. The dashed lines show 95% confidence interval. In Table 3 the numerical values and definitions of each parameter were summarized. The results show that all parameters are structurally identifiable, *i.e.* they have a finite limit.

The final model (M0) was fitted to the estimation data, see Fig. 4A–C, using a standard chi-square cost function as optimization objective function (see Materials and Methods). The solid curves in Fig. 4A–C correspond to predictions made with the parameters yielding the lowest cost (Eq. 2), and the dashed lines correspond to the most extreme predictions of MO yielded by the PL parameter confidence intervals (Table 3, Fig. 5). The corresponding predictions for a rejected model (M7) is shown for comparison in Fig. 4D–F, this model failed the chi-square test (Table 4).

**Table 3:** Estimated parameter values.

Parameter	Estimated value	PL-based confidence intervals	Unit	Description
ξ	1.604	[1.431; 1.801]		Scaling
*k_hb_*	3.852e-4	[2.108e-4; 6.657e-4]	s^−1^	Flux; from the hepatocyte to the bile.
*k_diff_*	1.731e-3	[9.626e-4; 2.915e-3]	s^−1^	Diffusion constant; between the EES and the blood plasma
*k_ph_*	4.776e-3	[3.265e-3; 6.682e-3]	s^−1^	Flux; from the plasma to the hepatocyte
*k_hp_*	2.857e-4	[4.593e-6; 6.754e-4]	s^−1^	Flux; from the hepatocyte to the plasma

Estimated parameter values and the associated profile likelihood-based (PL) confidence intervals for model M0, the PL estimate is shown graphically in Fig. 5.

**Table 4:** Model selection and testing process summary.

Model	No of fitted parameters	No. of identifiable parameters^a^	Fits the estimation data?	Lowest cost for estimation data (Eq. 2)^b^	Passes the *ad hoc* constraint?	Fits the validation data
M0	5	5	Yes	16.2 (20.3)	Yes	Yes
M1	6	3	Yes	16.2 (21.9)	Yes	Yes
M2	4	-	Yes	16.2	No	-
M3	6	-	Yes	20.5	No	-
M4	5	-	Yes	16.2	No	-
M5	4	-	No	332	-	-
M6	4	-	No	28.6	-	-
M7	5	-	No	63.1	-	-
M8	8	3	Yes	16.2 (21.9)	Yes	Yes
M9	6	-	Yes	16.2	No	-

a As defined by the PL-analysis.

b The cut-off for the chi-square test was 23.684. The value in the parenthesis is the lowest cost for passing the *ad hoc* constraint.

Summary of the model selection process, where the too simplified models fail to match the estimation data (M5–M7) and the *ad hoc* constraint (M2–M5, and M9) – that there is at least 1% of the tracer residing within the blood plasma pool after 3 h. A summarized description of the different rejected model variants are found in Fig. 3 and the suggested model, M0, in Fig. 2. There are only 3 models (M0, M1, and M8) which are able to pass the *ad hoc* constraint. Moreover M0, M1, and M8 successfully pass all the validation steps. The differences between M0 and the other two model variations (M1 and M8) are nonlinearities (Michaelis-Menten kinetics, see Fig. 3), which affects the number of practically identifiable parameters.

The same M0-parameters (as in Fig. 4A–C) were used for Fig. 4G, which displays the plasma concentration as a function of time. Certain parameter sets results in dose-normalized plasma concentrations at t = 3 h that were below 1% of the administered dose. Such a low concentrations below what is physiologically realistic [8], [33], suggests that even more parameters should be rejected. We therefore added an additional condition that the dose-normalized serum concentration at t = 3 h must be >1%. The parameters that fulfilled this additional fixed-limit requirement were used further below.

### Identifiability

**Figure 6:**
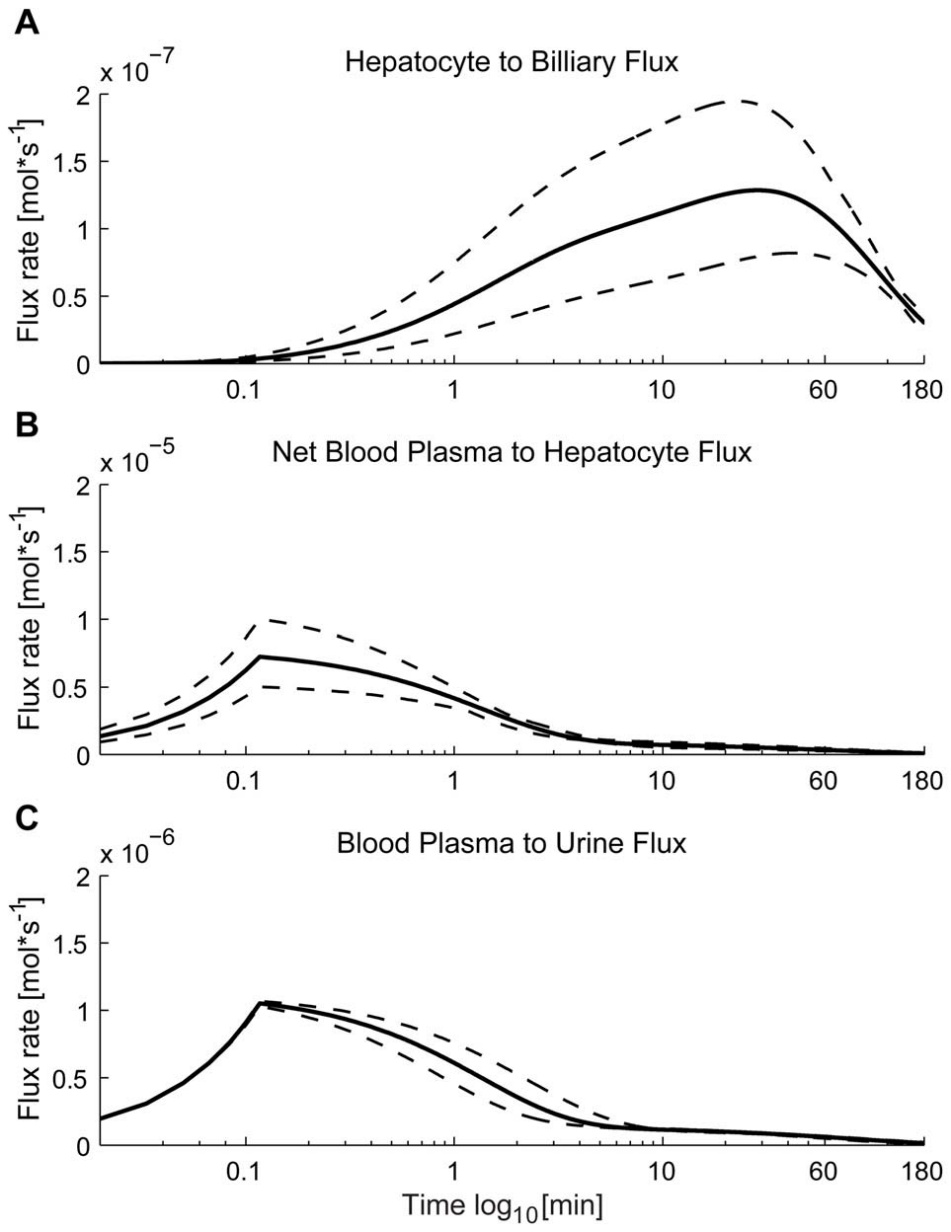
Contrast agent flux rates for the proposed model. The predicted CA fluxes in model M0 for; CA elimination via the liver (panel A), net Blood Plasma to Hepatocyte CA flux (panel B), and the elimination of CA via the kidneys (panel C). The dashed lines indicate the most extreme predictions defined by the profile likelihood-based confidence intervals of the parameters (Table 3).

A important strength of the model is that it is identifiable, meaning that the parameter values were determined by the experimental data with a low uncertainty [24]. This identifiability was analyzed using the PL-method ([24], see also Materials and Methods), and an example of such an analysis for model M0 is shown in Fig. 5. The determined parameter ranges for the fitted parameters are summarized in Table 3 and the effects on the rate equations can be appreciated in Fig. 6.

### Deriving a Minimal Model

Another important property of the model is that it is minimal given these data and requirements. This property is illustrated in Table 4, which shows the model properties for a number of different reasonable modifications or extensions of this model. The different versions corresponded to simplifications such as excluding certain fluxes (in M2–8), or by incorporating nonlinearities such as Michaelis-Menten expressions for describing some of the fluxes (M1 and M9) in an appropriate kinetic meaningful fashion (see Fig. 3). Our analysis showed that most of the in such a way simplified or extended models were not able to describe the data in a fully identifiable or statistically acceptable manner (as shown in Fig. 4C–F for M7). Furthermore, the models with introduced non-linear characteristics had at least three unidentifiable parameters (Table 4).

### Validation of the Minimal Model

**Figure 7:**
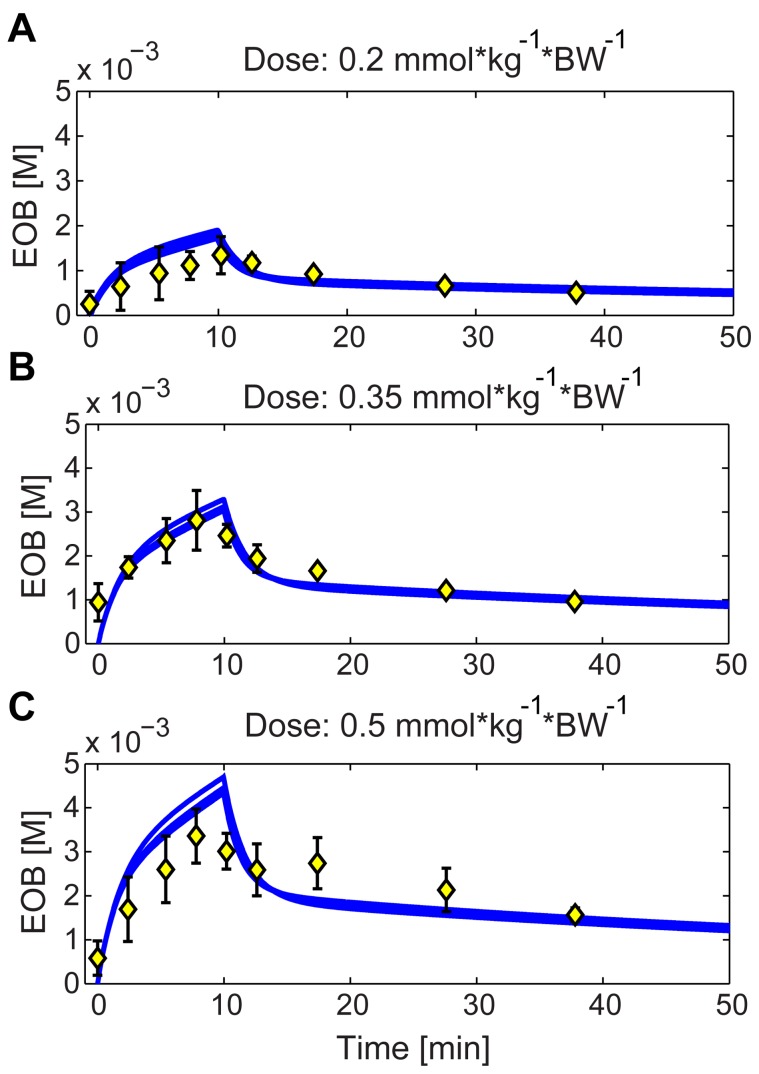
Model blood plasma predictions versus validation data. Model predictions of the blood plasma CA concentrations made using model M0, versus validation data (error bars corresponding to mean ± one standard deviation). Panels A–C corresponds to doses 8, 14, and 20 times higher than the clinically normal dose which is, 0.025 mmol·kg^−1^·BW^−1^ (which was used for the estimation data). The blue lines are model predictions obtained using the same set of parameters in all three cases. These were derived from the estimation data and the arbitrary scaling constant was then re-calculated for this data set, but kept equal in all three cases (A–C). The parameters of the input function were modified to mimic the experimental setup of the study. A chi-square test was used to test for differences.

A final strength of the minimal model is that it allows the description of independent validation data, something that was not used in the estimation phase. Here, two different such validation tests have been performed. First, we tested that the relative amounts of CA excreted to the bile and to the urine were within reasonable limits [8], [33], and the results are presented in Table 5. Second, the model was compared with three different datasets using doses ranging up to 20 times higher than those used in clinical practice and in the estimation data (Fig. 7A–C). As can be seen by visual inspection, the model agrees reasonably well with the validation data. Moreover a chi-square test confirmed this statistically.

**Table 5:** Predictions of renal and biliary elimination.

	Estimation data			Reference values	
	Predictions from the initial model fit	Predictions after applying the *ad hoc* constraint	Predictions based on the PL-analysis results	Data are shown as mean and SD	
**Bile**	38.4–75.4%	43.2–44.5%	38.4–63.5%	31±17% [33]	36.8±8.5% [8]
**Urine**	23.0–40.6%	39.0–39.7%	30.6–40.6%	48±5% [33]	43.6±8.6% [8]

Renal and biliary elimination fractions, expressed in per cent of administered dose, using model M0. The table shows how better defined the predictions become once the *ad hoc* constraint is applied or the PL-analysis (which includes the *ad hoc* constraint modified into a data point), in comparison to the predictions based on the sets of parameter derived from the estimation data. In the rightmost column some reference values for the EOB elimination are shown for comparison.

### Predictions

The model predications based on a representative set of parameter vectors given the estimation data for model M0 are shown in Fig. 6, with uncertainties using the core-prediction methodology (see Materials and methods). Moreover the predictions of the EOB elimination (via the renal or biliary pathways) are presented in Table 5, the increased prediction certainty on the eliminations due to the *ad hoc* constraint on the blood plasma concentrations is clearly shown in Table 5.

**Figure 8:**
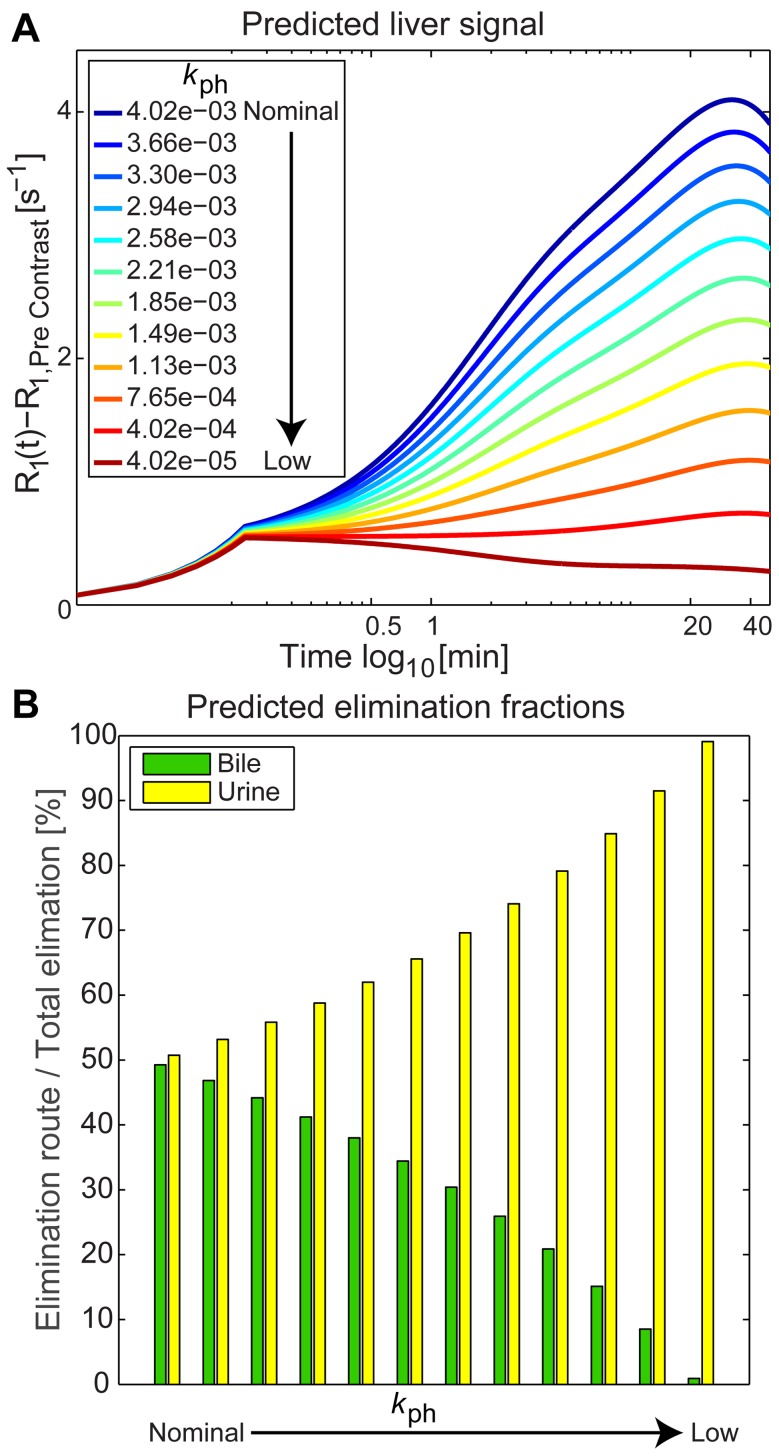
Simulated loss of liver function. A gradual loss of liver function was simulated using a set of parameter vectors based on the optimal values, but with reduced uptake kinetics. See the legend in panel A. The topmost, dark blue, line corresponds to the optimal values from Table 3. The predicted liver signals obtained using model M0 are shown in Panel A, and also the corresponding predicted elimination fractions in panel B (left to right on the x-axis). The elimination fractions were calculated as the individual route divided by the total elimination for the simulation (bile or urine, green and yellow bar respectively).

To further evaluate the soundness of M0, the model was simulated for a new set of parameter vectors based on the optimal parameter values (Table 3) with a decreasing hepatocyte uptake (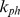) until a zero uptake was approached (all other parameters were fixed). Fig. 8A shows how the predicted liver signal then decreases as the uptake rate was lowered for rather narrow range until there was practically no uptake. Fig. 8B shows the predications for how this reduced contrast agent uptake affected the elimination routes.

### Sensitivity of the liver signal due volume variations

**Figure 9:**
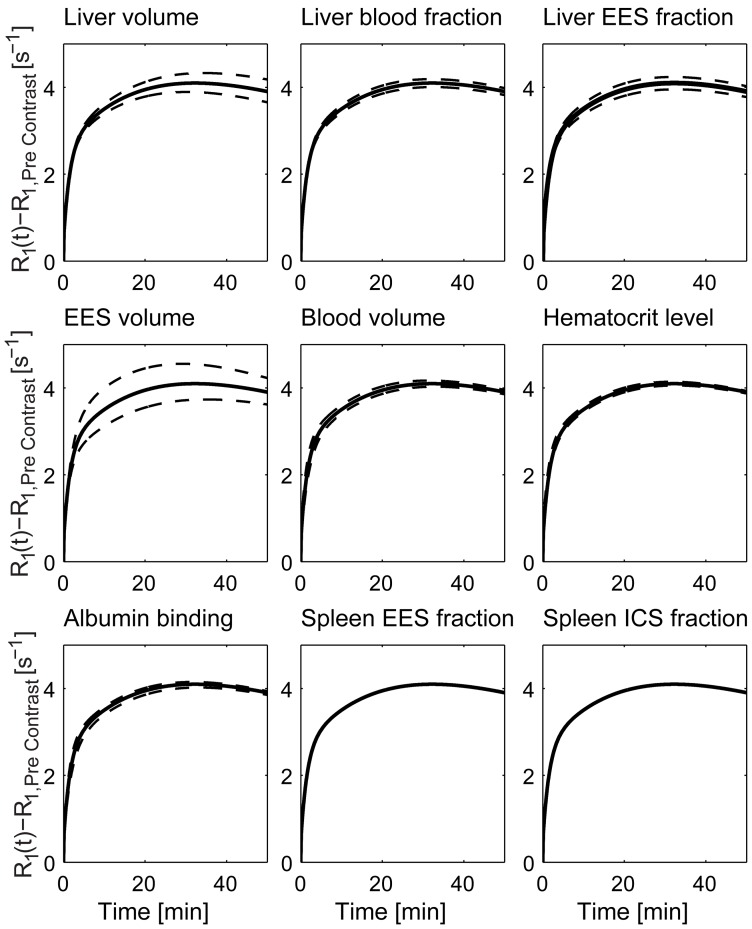
Prediction sensitivity due to volume variations. The final model, M0, was simulated using variable volume parameter values, and each volume parameter was allowed to differ ±20% from its nominal value, together with the optimal rate parameter values (Table 3). The solid black lines correspond to the predicted liver signal using the nominal values, whereas the dashed lines shows the sensitivity of the predicted liver signal due the volume variations. Each panel corresponds to a specific volume parameter as indicated by the title above it. M0 is most sensitive to variations in the volume parameters corresponding to; EES volume, liver volume and liver EES fraction.

In the sensitivity analysis, the final model (M0) was found to be primarily sensitive to changes in the whole liver and EES volume parameters, as shown in Fig. 9, where each volume parameter was allowed to have a value of ±20%.

## Discussion

We have presented a new modeling approach, which constitute a biologically highly relevant data-analysis for DCE-MRI, and exemplified it using EOB uptake in human liver in combination with a clinical protocol. The main benefit of our approach is that it estimates realistic kinetic transport parameters, describing liver function. These parameters are extracted through modeling of dynamic MRI-measurements that are normally interpreted by radiologists, although such transport parameters would not be available using such routine clinically oriented visual inspection. Recent work has indicated that quantifying such spatial highly resolved DCE-MRI images can be used for discrimination between fibrosis grade [11] and impaired hepatobiliary function [10]. Our presented model is minimal, identifiable, and provides physiologically realistic predictions.

There are some specific strengths with our approach. First, the parameter uncertainty was determined using a novel method, PL [24], which is more correct and generally applicable than the more widely used approaches based on the Hessian of the cost function [41]. Second, that our approach has yielded an identifiable model means that it is also observable, *i.e.* all model predictions will have a both well-defined and small uncertainty. Third, access to well-defined parameters means that there is a potential that some of them likely can be replaced by their determined values in future clinical/model work, leading to a suitable simplification. For instance, the *k_Diff_* parameter is clearly both well-characterized and well-defined, and it is also not expected to change in diseased states. Thus, replacing this parameter with estimated determined realistic value would potentially enhance the identifiability on other diagnostically relevant parameters in future versions of this model. Fourth, we have used an approach based on *ad hoc* constraints, in order to eliminate physiologically unrealistic parameters. This approach turned out to be successful, since we obtained realistic values for non-rejected parameters for other model properties (*e.g.* the more well defined elimination fractions, see Table 5 and Fig. 7) than those, which were used for the original constraint. Such *ad hoc* constraint should also be useful in the case of hepatic and/or renal impairment, as shown in [33]. A few final comments regarding the strength of the model is that the same model parameters were able to describe a dose that is 20 times higher than the clinically used CA-dose, and administered using 10 min infusion instead of 7 s bolus injection in the estimation data (the input function's parameters where changed to match the experimental setup, Eq. 3). This indicates a remarkable robustness of the model that is useful in future work. Moreover since the model is physiologically realistic and identifiable, the model is expandable, meaning that new information can be added into the framework such as other relevant observations (*e.g.* renal clearance, volumetric MRI, and additional ROIs such as bile) and detailed sub-models. This added information will improve the predictions made by the model, as already illustrated by the fact that inclusion of the *ad hoc* constraint on the blood plasma yields more accurate CA elimination fractions.

When interpreting the minimal model and the results, there are a few underlying assumptions and limitations that should be kept in mind. First, there is an initial wash-in phase connected (≈1 min) with the bolus injection of CA, when the injected CA has not yet been distributed evenly in the blood, and this initial wash-in is not accurately described by our present model(due to the present formulation of ODEs which imply a instantaneously mixed compartment). The limitation leads to a slightly higher uncertainty in the first data point which therefore possibly should be corrected for in future work. Second, we have used some parameter values from the literature. For instance, we have obtained tissue volumes and tissue composition parameters from a ‘standard human’. A more sophisticated approach would estimate these parameters in each individual while collecting experimental data, as can be seen in Fig. 9 the model is influenced by variations in the volume parameters relating to the whole liver volume and EES volumes, the first of which is easily quantified from suitable images. Based on whole body scans, we believe that accurate EES volumes should also be possible to derive. Third, it would be beneficial to include measurements of the common bile duct, in order to estimate the biliary excretion more accurately. However, at this time we have not been able to obtain sufficiently reliable values in measurements. Fourth, the *ad hoc* could not be implemented directly in PL-analyses; hence it was included as a new data-point which leads to a larger parameter uncertainty than would otherwise be expected (should the constraint be applied strictly). Fifth, the elimination fractions presented in Table 5, compared to the reference values, should be evaluated with care since there are differences in; gender distribution, temporal differences (6 days in the reference data, 3 h in the simulations), slight differences in doses, and finally we have not implemented recirculation of EOB from the intestine in the model which to an extent would increase the amount of EOB eliminated via the bile. Sixth, it is assumed that there is no enterohepatic circulation of EOB, however, earlier studies on rats have indicated that this possibility of reabsorption into the blood can be excluded which we assumed was a reasonable assumption in humans too [42]. Moreover, if such reabsorption would exist in humans the effect on the EOB concentrations (in *e.g.* the plasma fraction of the blood stream) would be completely negligible within the time frame of the DCE-MRI examinations. Finally, a long-term and very obvious goal with the approach described here is to use it for regional liver function estimation in a clinical context, but this would require more advanced post-processing registration techniques, and we have therefore used simple ROIs in this work to demonstrate what we hope will be a highly useful clinical procedure in the near future.

It should also be noted that we do not in any way dispute the use of Michaelis-Menten kinetics for describing the particular transfer of CA from the hepatocytes into the bile by MRP2. Rather within the context of this work in terms of the data available and with our aim to find a minimal and identifiable system, we found that 1) we can describe the system at least equally well using mass action based kinetics, 2) the nonlinearities and extra parameters that unavoidably arise as a consequence of the use of Michaelis-Menten kinetics significantly reduced the amount of identifiable parameters. Clearly this is very advantageous when developing clinically relevant procedures. Possibly a higher temporally resolved data set would allow for such a description, which was used in [15], and in Model M1 in this work for comparison.

In conclusion, this work constitutes in our opinion an important step towards both quantitative and non-invasive regional liver function estimation in human subjects, and therefore clearly also a clinically useful future procedure. The model represents the entire system that is proved by the CA, and it is minimal and identifiable, which means that clinically interesting kinetic parameters and fluxes now readily can be identified. To our knowledge this is the first whole body physiologically based mechanistic model. Moreover, the framework allows for using both highly spatially and low temporally resolved data, which means that suitably processed data can also be used for visual assessment by a radiologist at a great level of detail *i.e.* regional function assessment. We therefore believe that the described procedure will contribute to a much better evaluation of hepatic function than what is presently available to the clinician, *e.g.* in characterizing early disease stages or in pre-operative planning of respective surgery of the liver.

## Supporting Information

File S1 **Scripts and models.** This file contains all relevant model definitions and scripts used in this work.
(TGZ)
