# Correction: Physiologically Realistic and Validated Mathematical Liver Model Revels Hepatobiliary Transfer Rates for Gd-EOB-DTPA Using Human DCE-MRI Data

**DOI:** 10.1371/journal.pone.0104570

**Published:** 2014-07-31

**Authors:** The PLOS ONE Staff

The word “Reveals” is misspelled in the article title. The correct title is: Physiologically Realistic and Validated Mathematical Liver Model Reveals Hepatobiliary Transfer Rates for Gd-EOB-DTPA Using Human DCE-MRI Data. The correct citation is: Forsgren MF, Leinhard OD, Dahlström N, Cedersund G, Lundberg P (2014) Physiologically Realistic and Validated Mathematical Liver Model Reveals Hepatobiliary Transfer Rates for Gd-EOB-DTPA Using Human DCE-MRI Data. PLoS ONE 9(4): e95700. doi:10.1371/journal.pone.0095700
