# Supplementary material for: Physiologically Realistic and Validated Mathematical Liver Model Revels Hepatobiliary Transfer Rates for Gd-EOB-DTPA Using Human DCE-MRI Data
Source: PLoS One. 2014 Apr 18;9(4):e95700. doi: 10.1371/journal.pone.0095700 (PMC3991717; doi:10.1371/journal.pone.0095700)
Supplement: File S1 — Scripts and models. This file contains all relevant model definitions and scripts used in this work. (TGZ) [file pone.0095700.s001.tgz › Supplementary/README.rtf]

The following functions and scripts are located in each model folder (e.g. M0), except when it was not deemed necessary since the model was falsified in an earlier test (compare with Table 4).###MEX-compilation of the model(s):- compileModelsModel optimization scripts:	-	optimScript	The main script which initiates the parameter optimization, and sets all settings for 	the sub-functions.	-	costFunction	Objective function for the optimization, preforms the chi2-test.	-	simannealingSBAOClusteringL	The optimization algorithm.	-	getSpreadedParam	Gets the most extreme parameter values (and the associated parameter vectors) 	for a quick post-optimization plot of the results.	-	plotFunction	Plots various aspects of the model after the optimization.Ad hoc constraint:	-	adhocSim	Applies the ad hoc constraint to a given set of parameter vectors.Validation:	-	validateParam	Checks whether the sets of parameter vectors can fit the validation data.Profile likelihood:	-	runPL	Short script with settings for initiating the PL-analysis.	-	costFunctionPL	Objective function for the PL-analysis.	-	raueParamEst	Main PL-function.###Article plot functions (separate folder):	-	plotElim	Calculates the elimination fractions (Table. 5).- plotPredNPL	Plots the model predictions vs experimental data as well as the PL-analysis results 	(Fig. 3 & Fig.4).	-	plotValid	Plots model predications vs the validation data (Fig. 5).	-	plotFlow	Plots interesting flows in the model (Fig. 6).
